# Spontaneous feline mammary intraepithelial lesions as a model for human estrogen receptor- and progesterone receptor-negative breast lesions

**DOI:** 10.1186/1471-2407-10-156

**Published:** 2010-04-22

**Authors:** Giovanni P Burrai, Sulma I Mohammed, Margaret A Miller, Vincenzo Marras, Salvatore Pirino, Maria F Addis, Sergio Uzzau, Elisabetta Antuofermo

**Affiliations:** 1 Department of Pathology and Veterinary Clinic, Faculty of Veterinary Medicine, Sassari University, Italy; 2 Department of Comparative Pathobiology and Animal Disease Diagnostic Laboratory, Purdue University, West Lafayette, IN 47907, USA; 3 Institute of Anatomy and Histopathology, Sassari University School of Medicine, Sardinia, Italy; 4 PortoConteRicerche Srl, Tramariglio, Alghero, Sassari, Italy; 5 Purdue Cancer Center, Purdue University, West Lafayette, IN 47907, USA; 6 Bindley Bioscience, Discovery Park, Purdue University, West Lafayette, IN 47907, USA

## Abstract

**Background:**

Breast cancer is the most frequently diagnosed cancer in women. Intraepithelial lesions (IELs), such as usual ductal hyperplasia (UH), atypical ductal hyperplasia (ADH), and ductal carcinoma in situ (DCIS) are risk factors that predict a woman's chance of developing invasive breast cancer. Therefore, a comparative study that establishes an animal model of pre-invasive lesions is needed for the development of preventative measures and effective treatment for both mammary IELs and tumors. The purpose of this study was to characterize the histologic and molecular features of feline mammary IELs and compare them with those in women.

**Methods:**

Formalin-fixed, paraffin-embedded specimens (n = 205) from 203 female cats with clinical mammary disease were retrieved from the archives of the Purdue University Animal Disease Diagnostic Laboratory and Veterinary Teaching Hospital (West Lafayette, IN), and the Department of Pathology and Veterinary Clinic, School of Veterinary Medicine (Sassari, Italy). Histologic sections, stained with hematoxylin and eosin (HE), were evaluated for the presence of IELs in tissue adjacent to excised mammary tumors. Lesions were compared to those of humans. Immunohistochemistry for estrogen receptor (ER-alpha), progesterone receptor (PR), human epidermal growth factor receptor 2 (HER-2/neu) and Ki-67 was performed in IELs and adjacent tumor tissues.

**Results:**

Intraepithelial lesions were found in 57 of 203 (28%) feline mammary specimens and were categorized as UH (27%), ADH (29%), and DCIS (44%). Most IELs with atypia (ADH and DCIS) were associated with mammary cancer (91%), whereas UH was associated with benign lesions in 53% of cases. Feline IELs were remarkably similar to human IELs. No ER or PR immunoreactivity was detected in intermediate-grade or high-grade DCIS or their associated malignant tumors. HER-2 protein overexpression was found in 27% of IELs.

**Conclusion:**

The remarkable similarity of feline mammary IELs to those of humans, with the tendency to lose hormone receptor expression in atypical IELs, supports the cat as a possible model to study ER- and PR-negative breast lesions.

## Background

Breast cancer is the second leading cause of death in women in the United States, with about 1,479,350 expected cases in 2009. This accounts for 27% (192,370) of all new cancer cases among women and 562,340 deaths per annum [1]. With the implementation of mammographic screening, great progress in early breast cancer diagnosis has been achieved. Nowadays, many women are diagnosed with preinvasive intraepithelial lesions (IELs). Approximately half a million breast IELs are diagnosed yearly; these include 360,000 cases of usual hyperplasia (UH), 60,000 of atypical ductal hyperplasia (ADH) [2], and 57,604 of ductal carcinoma *in situ *(DCIS) [1].

Detection and evaluation of IELs are used routinely to estimate a woman's risk of developing breast cancer and to aid physicians in designing optimal therapeutic strategies. It was postulated, based on epidemiological studies, that the risk for breast cancer ranges from 1.5-2, 4-5, and 8-10, respectively, for women diagnosed with UH, ADH, and DCIS [3,4]. Understanding the histopathological and molecular characteristics of the IELs will assist in elucidating the pathogenesis of breast cancer and identifying specific therapeutic targets [5]. Several transplantable or chemically induced rodent models have been developed to study human cancers, including osteosarcoma, melanoma, bladder and intestinal tumors, non-Hodgkin lymphoma and mammary tumors [6]. However, these models lack many aspects of human cancers [7]. An animal model that develops spontaneous mammary tumors that resemble human breast cancer in many aspects is needed [8,9].

Feline mammary carcinoma is similar to human breast cancer in the age of onset, incidence, histopathologic features, biologic behavior, and pattern of metastasis [9,10]. The annual incidence of feline mammary neoplasia was estimated at 12.8-25.4 per 100,000 female cats [11]. About 85% - 93% of feline mammary tumors are malignant, and there is little breed-associated predilection, except that Siamese cats appear to have a 2-fold increased risk. Mammary neoplasia has been reported to occur in cats from 9 months to 23 years of age (mean, 10 to 12 years) [11,12]. Hormonal influences are probably involved in the pathogenesis of feline mammary neoplasia. Cats that are ovariohysterectomized before 6 months or 1 year of age had 91% and 86% reduction in risk of developing mammary tumors, respectively, when compared to intact female cats [13].

These data implicate ovarian hormones in the development of feline mammary tumors [14]. The influence of hormonal factors is emphasized by Misdorp *et al.*, who demonstrated that the regular and prolonged administration of progestins (used for estrus prevention in cats) increased the risk of mammary tumor development [15]. The influence of ovarian hormones is also well-established in humans. Early menarche, before age 12, increases the risk four-fold, as does late menopause [16].

In humans, ER+ tumors have a better prognosis, and 50-60% of cases tend to respond to hormonal treatments [17,19]. ER+ breast carcinomas are usually (70-80%) well-differentiated with low expression of proliferation markers [18]. However, 30% of human breast cancers are ER-negative with a worse prognosis than ER-positive tumors [19]. Most cats (80%) tend to have ER-negative, highly aggressive mammary tumors; thus, they may be particularly suitable as animal models of human hormone-unresponsive breast cancer [20].

Human epidermal growth factor receptor 2 (HER-2/*neu*) is a cell-membrane receptor tyrosine kinase, normally involved in the signal transduction pathways leading to cell growth and differentiation [21]. Approximately 15-20% of breast cancers have amplification of the HER-2/*neu *gene or over-expression of its protein product. HER-2/*neu *protein over-expression is associated with increased disease recurrence and has been used to predict patient response to treatment [22]. De Maria *et al. *reported that HER-2 gene kinase domain in cats and humans has 92% homology [23]. HER-2 protein was highly expressed in feline carcinomas when compared to human breast carcinoma, suggesting its possible role as a prognostic marker [24,25].

To the best of our knowledge, feline mammary IELs have not been compared with pre-invasive lesions of the human breast. Thus, this study was undertaken to investigate the prevalence and types of IELs in feline mastectomy specimens and compare them to human breast IELs, and to determine the expression of ER-α, PR, HER-2/*neu*, and Ki67 by immunohistochemistry.

## Methods

### Tissue samples

Two hundred five formalin-fixed, paraffin-embedded specimens from 203 female cats with clinical mammary disease were retrieved from the archives of the Purdue University Animal Disease Diagnostic Laboratory and Veterinary Teaching Hospital (West Lafayette, IN) and the Department of Pathology and Veterinary Clinic, School of Veterinary Medicine, (Sassari, Italy). Eighty cats had been spayed before diagnosis; 122 were sexually intact; the sexual status of 1 cat was unknown. The cats' age ranged from 0.5-18 years (median, 10 years). Cats of different breeds were included (82 domestic shorthair, 18 domestic long-hair, 21 European, 10 Siamese, 8 Persian, 4 mixed-breed, 1 Himalayan, 1 Burmese); 58 cats had no breed designation.

### Histology

Histologic sections, stained with hematoxylin and eosin (HE), were evaluated for the presence of IELs in tissue adjacent to excised mammary tumors. Lesions were classified according to criteria for IELs of the human breast [26,27]. In this study, we focused on the best-characterized IELs that arise in the terminal duct-lobular units; these included UH, ADH and DCIS (low-, intermediate-, and high-grade) [5].

Lesions were classified in consultation with an MD pathologist (VM) and compared with IELs in women. Human samples were obtained from the Institute of Anatomy and Histopathology, Sassari University School of Medicine. The study protocol was approved by the Ethical Committee at the University of Sassari. Features applicable to usual ductal hyperplasia (UH), also called epitheliosis, consisted of ducts partially filled by a mixed population of epithelial and myoepithelial cells that exceeded 3 or 4 layers in thickness. The diagnosis of atypical ductal hyperplasia (ADH) was made when the mixed population of epithelial and myoepithelial cells had nuclear atypia. In some cases, cords of epithelial cells formed bridges with irregular fenestrations. In IELs in which cytologic features of low- or intermediate-grade DCIS were observed, but confined to 1 duct cross-section, the IEL was classified as ADH [26,28]. Ductal carcinoma *in situ *(DCIS) was diagnosed when the IEL was composed purely of epithelial cells with cytologic and architectural atypia. Based on these cytological and architectural characteristics, DCIS was subdivided into 3 categories. Low-grade DCIS was composed of a proliferation of monomorphic cells with hyperchromatic central nuclei, inconspicuous nucleoli and few mitotic figures. Intermediate-grade DCIS was distinguished by the lack of the monotonous aspect and moderate nuclear pleomorphism. Finally, high grade DCIS was composed of pleomorphic atypical cells with large nuclei, prominent nucleoli, and frequent and/or atypical mitotic figures. Different patterns were observed (cribriform, papillary, micropapillary, solid, and solid with comedo-type necrosis). Tumors were classified according to WHO Histological Classification of Mammary Tumors of the Dogs and Cats [29] and graded according to a semi-quantitative scheme, originally developed in humans [30] and applied to feline mammary carcinoma by Castagnaro [31]. Mammary carcinomas were graded as well (WDC), moderately (MDC) and poorly differentiated (PDC) carcinoma. Percentage of tubule formation, mitotic index, cellular and nuclear morphology were each assigned an individual score from 1 to 3 and then added, classifying carcinoma as follows: grade I (WDC), 3-5 points; grade II (MDC), 6-7 points; grade III (PDC), 8-9 points.

### Immunohistochemistry

Immunohistochemistry was performed using the labeled streptavidin biotin (LSAB) method. Histologic sections (5 μm thick) from formalin-fixed, paraffin-embedded feline mammary tissue with IELs and without IELs (control tissue) were mounted on positively charged Superfrost slides (Fisher Scientific). Tissue sections were deparaffinized and rehydrated through a series of graded alcohols. Antigens were retrieved by a high-temperature heating method (slides were immersed in target retrieval solution at pH 6 [Dako Cytomation], in a steamer (90-95°C) with a 20-minute incubation for all antigens except for Her-2 *neu*, for which slides were kept in a water bath for 40 min at 97°C. Tissues were then blocked for endogenous peroxidase in 3% hydrogen peroxide in water, and for nonspecific binding in PBS containing 0.25% casein, stabilizing protein and 0.015 mol/L sodium azide (Protein Block Serum-Free, DakoCytomation). Tissues were incubated overnight at 4°C in the following antisera: ER-α monoclonal mouse anti-human antibody clone NCL-ER-6F11 at 1:40 dilution (Novocastra Ltd.), progesterone receptor monoclonal antibody PR 10A9 at 1: 50 dilution (Immunotech, Marselle, France), Ki67 monoclonal mouse anti-human antibody clone MIB-1 at 1:50 dilution (DakoCytomation), and HER-2/*neu *polyclonal rabbit anti-human antibody at 1:100 dilution (DakoCytomation), followed by biotinylated goat anti-mouse or goat anti-rabbit secondary antibodies (DakoCytomation). The chromogen was 3,3'-diaminobenzidine (DakoCytomation). Sections were counterstained with Mayer's hematoxylin and then cover-slipped in 50:50 xylene/Permount (Fisher Scientific). Negative control slides were treated with isotype-matched IgG serum. Control slides, known to be positive for each antibody, were incorporated into each run. Nuclear immunostaining for ER, PR and Ki-67 was evaluated counting a total of 1000 cells in 10 representative fields at high magnification (400×) whereas for smaller lesions, the entire lesion was considered. The number of immunopositive cells was expressed as a percentage (mean, median, minimum and maximum values).

The intensity of ER, PR, and Ki67 immunoreactivity was graded on a scale of 0 to 3, in which 0 = no reactivity, 1 = weak, 2 = moderate, and 3 = strong reactivity.

The over-expression of HER-2/*neu *was defined as increased cell membrane reactivity of epithelial cells. The scoring system according to the HercepTest™ can be summarized as follows: 0 = no staining or weak and incomplete membrane staining in less than 10% of the neoplastic cells; 1+ = incomplete and faint membrane staining in more than 10% of the neoplastic cells; 2+ = moderate and complete membrane staining in more than 10% of the neoplastic cells; 3+ = strong and complete membrane staining in more than 10% of the neoplastic cells. Scores of 0 or 1 were considered negative, whereas 2 or 3 were considered positive for HER-2/*neu *over-expression.

### SDS-PAGE and Western Immunoblotting

Protein was extracted from fresh-frozen biopsy specimens from 5 feline mammary carcinomas and 5 normal mammary glands. Liver was used as a control. Replicate 5-μm-thick slices were cut from frozen tissue blocks. Five sections of each sample were placed in 2-mL Eppendorf safe-lock tubes and immersed in Laemmli buffer for lysis. After incubation on ice for 20 min, tissue lysates were clarified for 10 min at 12,000 × g at 4°C, denatured at 95°C for 5 min, and stored at -80°C until needed. For electrophoresis, protein extracts from fresh-frozen mammary and liver specimens were subjected to SDS-PAGE in 8% polyacrylamide gels according to Laemmli [32]. Electrophoresis was stopped when the tracker dye reached the end of gels. Proteins were then stained with Coomassie Brilliant Blue R 250 (Sigma-Aldrich, St. Louis, MO) according to Westermeier [33], decolorized and digitized with an Image Scanner (GE Healthcare).

For western immunoblots, electrophoresed proteins were transferred to nitrocellulose membranes and blocked in phosphate buffered saline, 0.05% Tween 20 (PBS-T), plus 5% skim milk for 1 hour to overnight. The membrane was then incubated with the HER-2/*neu *polyclonal rabbit anti-human antibody at 1:1000 dilution (DakoCytomation) in PBS-T plus 2% skim milk for 2 hours, washed five times with PBS-T, and incubated for 1 h with peroxidase-conjugated goat anti-rabbit secondary antibodies (Sigma-Aldrich) in PBS-T plus 2% skim milk. After washing the membrane five times with PBS-T, immunoreactivity was visualized by incubation with a chemiluminescent peroxidase substrate (Sigma-Aldrich).

### Statistical analysis

Differences in IHC expression of ER, PR, Her-2/*neu *and Ki67 between types of IELs (e.g., ADH and UH) was obtained by a standard t-test for two-group comparison. Correlation between IELs and the adjacent tumors for ER, PR, HER-2/neu and Ki67 was obtained by simple regression analysis.

## Results

### Histology

Sixty-three mammary IELs were identified in mastectomy specimens from 57 (28%) of the 203 female cats; 6 cats had multiple IELs. The lesions were classified as UH (n = 17, 27%), ADH (n = 18, 29%) and DCIS (n = 28, 44%), and are listed in Table 1, in order of increasing risk for the development of mammary carcinoma, as described in humans [3,4]. The association between IELs (UH, ADH, or DCIS) and mammary tumors is also illustrated. Different morphological patterns were observed in DCIS: 4 were cribriform (1 low grade, 3 intermediate grade), 9 papillary (2 low grade, 7 intermediate grade), 4 micropapillary (2 low grade, 1 low grade, 1 high grade), 6 solid (1 low grade, 1 intermediate grade, 4 high grade) and 5 were comedo type DCIS (5 high grade).

**Table 1:** Feline mammary IELs (classified by human risk [3,4]) and their association with invasive cancer

Risk	Number of IELs		% of total IELs	No. IELs associated with malignant tumors	% IELs associated with malignant tumors
**Slight**	Ductal hyperplasia n = 17		27%	8/17	47%

**Intermediate**	Atypical ductal hyperplasia n = 18		29%	17/18	95%

**High**	Ductal carcinoma *in situ* n = 28		44%	25/28	89%
	✓ low grade	6		6/6	100%
	✓ intermediate	11		9/11	82%
	✓ high	11		10/11	91%

**Figure 1:**
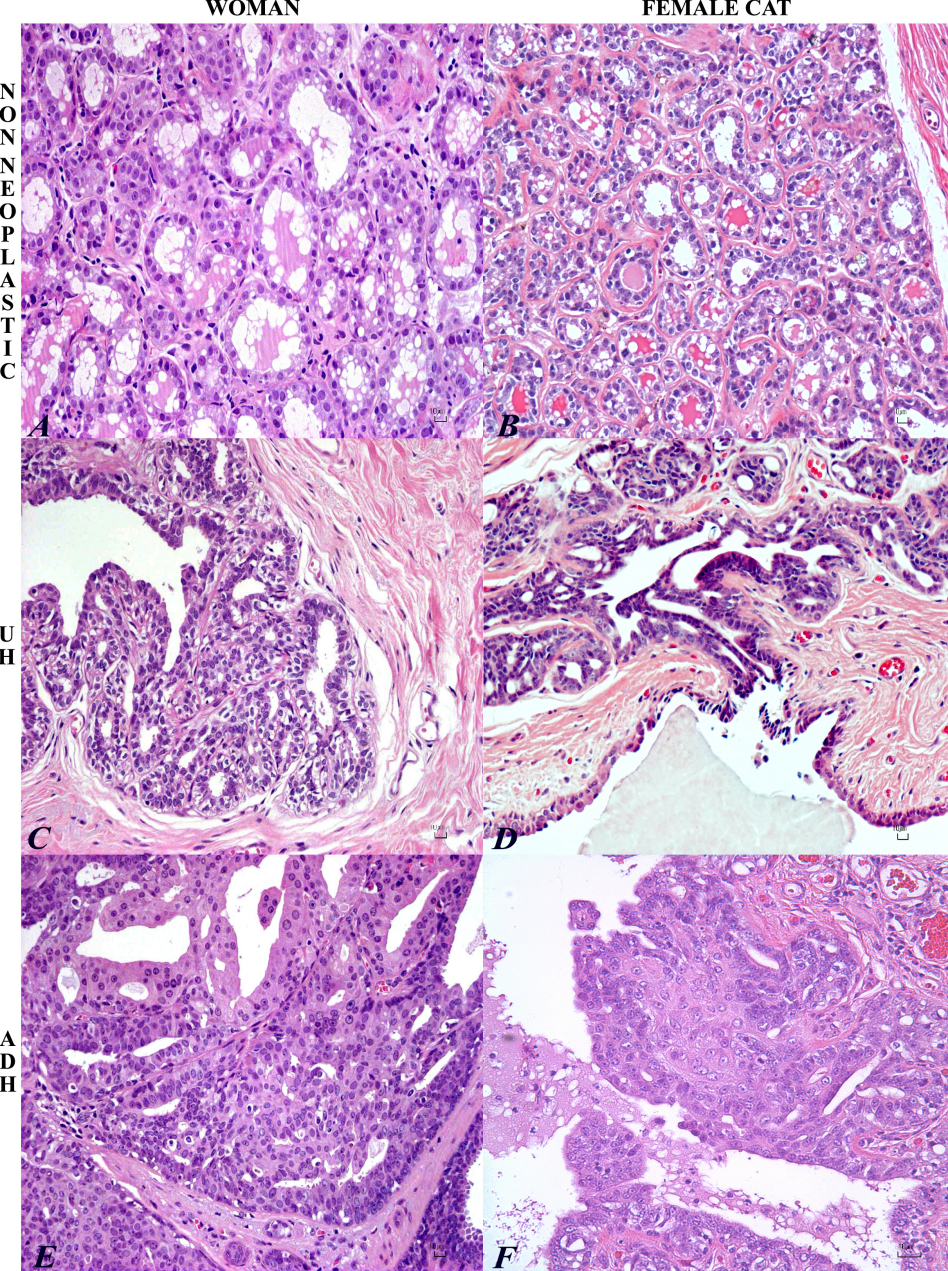
**Histopathology of normal mammary gland, UH, ADH in woman and cat**. Normal secretory mammary gland in woman (A) and cat (B): the epithelial cells are vacuolated with cytoplasmic accumulation of fat droplets. HE. Bar = 10 μm. Usual ductal hyperplasia (UH) in woman (C) and cat (D) with typical fenestrated growth pattern. The ducts are lined by monotonous luminal epithelial cells that are cuboidal to columnar, with hyperchromatic nuclei and rarely prominent nucleoli. Few myoepithelial cells are admixed in the hyperplastic epithelium. HE. Bar = 10 μm. Atypical ductal hyperplasia (ADH) in woman (E) and cat (F) with micropapillary projection of disorganized epithelial cells and spindle-shaped (myoepithelial) cells. Epithelial cells are enlarged with round nuclei with coarse chromatin and prominent single nucleoli. HE. Bar = 10 μm

**Figure 2:**
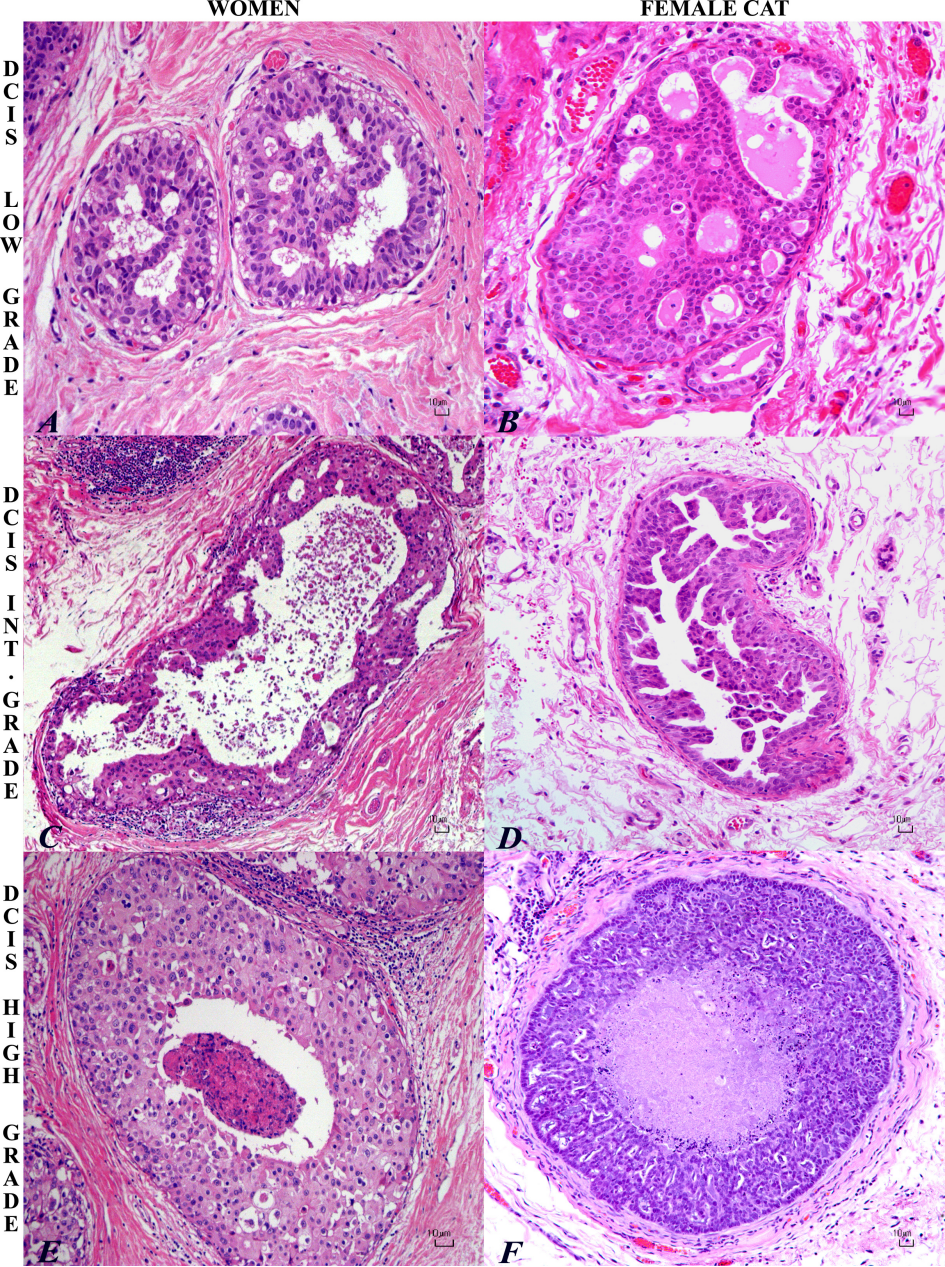
**Histopathology of low, intermediate, high-grade DCIS in woman and cat**. Low-grade ductal carcinoma *in situ *(DCIS) in woman (A) and cat (B) with proliferation of monomorphous, small and relatively uniform epithelial cells with moderate and eosinophilic cytoplasm, oval nuclei, arranged to form regular cribriform spaces. HE. Bar = 10 μm. Intermediate-grade ductal carcinoma *in situ *(DCIS) in woman (C) and cat (D) with proliferation of pleomorphic cuboidal cells with moderate eosinophilic cytoplasm, oval to elongate nuclei, and single prominent nucleoli; micropapillary pattern. Central secretion, necrotic cell debris and periductal inflammatory reaction is depicted in the human intermediate-grade DCIS. HE. Bar = 10 μm. High-grade comedo ductal carcinoma *in situ *in woman (E) and cat (F) with solid proliferation of epithelial cells in a distended duct with central necrosis. Highly pleomorphic cuboidal to oval cells with abundant eosinophilic granular cytoplasm, round vescicular nuclei and prominent nucleoli. Lymphocytes and plasma cells infiltrate the periductal stroma. Bar = 10 μm.

Eight of 17 (47%) cases of UH were associated with malignant tumors, whereas 9 of 17 (53%) cases of UH were associated with benign lesions. In contrast, 17 of 18 cases ADH were associated with malignant tumors and only one case of ADH (6%) was associated with a benign neoplasm. In addition, DCIS, sub-classified as low, intermediate, and high grade, was associated with malignant tumors in all except 3 cases. All IELs (UH, ADH, and DCIS), as well as non-lesional feline mammary gland, were histologically similar to their human counterparts as depicted in Figures 1 and 2. Of the 205 palpable mammary masses, 168 (82%) were malignant tumors. In addition, 21/205 (10%) benign tumors, 10/205 (5%) duct ectasia and 6/205 (3%) cases of fibroadenomatous change were identified. Table 2 lists the different histological patterns and grade of feline malignant mammary tumors. Six cases of tubulopapillary adenocarcinoma were sub-classified as micropapillary variant when more than 50% of the tumor had an infiltrating micropapillary pattern, as described by Seixas [34]. Based on the grading scheme, 14% of the mammary carcinomas were well differentiated; 51%, moderately; and 35%, poorly differentiated.

**Table 2:** Feline mammary tumors classified according to Elston and Ellis grading system [31]

*Histological pattern*	*totals*	*Number of tumors graded as*		
		
		Well differentiated	Moderately differentiated	Poorly differentiated
		(WDC)	(MDC)	(PDC)
Tubulopapillary carcinoma	123	24	76	23
Solid carcinoma	42	0	9	33
Squamous cell carcinoma	3	0	0	3

*totals*	168	24	85	59
%		14%	51%	35%

### Immunohistochemistry

Eight of 63 lesions could not be evaluated immunohistochemically either because of suboptimal fixation or lack of sufficient tissue in the paraffin block. Immunohistochemistry was performed on 55 IELs and associated tumor tissues, of which 44 were malignant. Twenty normal feline mammary gland, surrounding the 55 lesion, were also evaluated.

#### Expression of ER in IELs (Figure 3, A1-A6)

**Figure 3:**
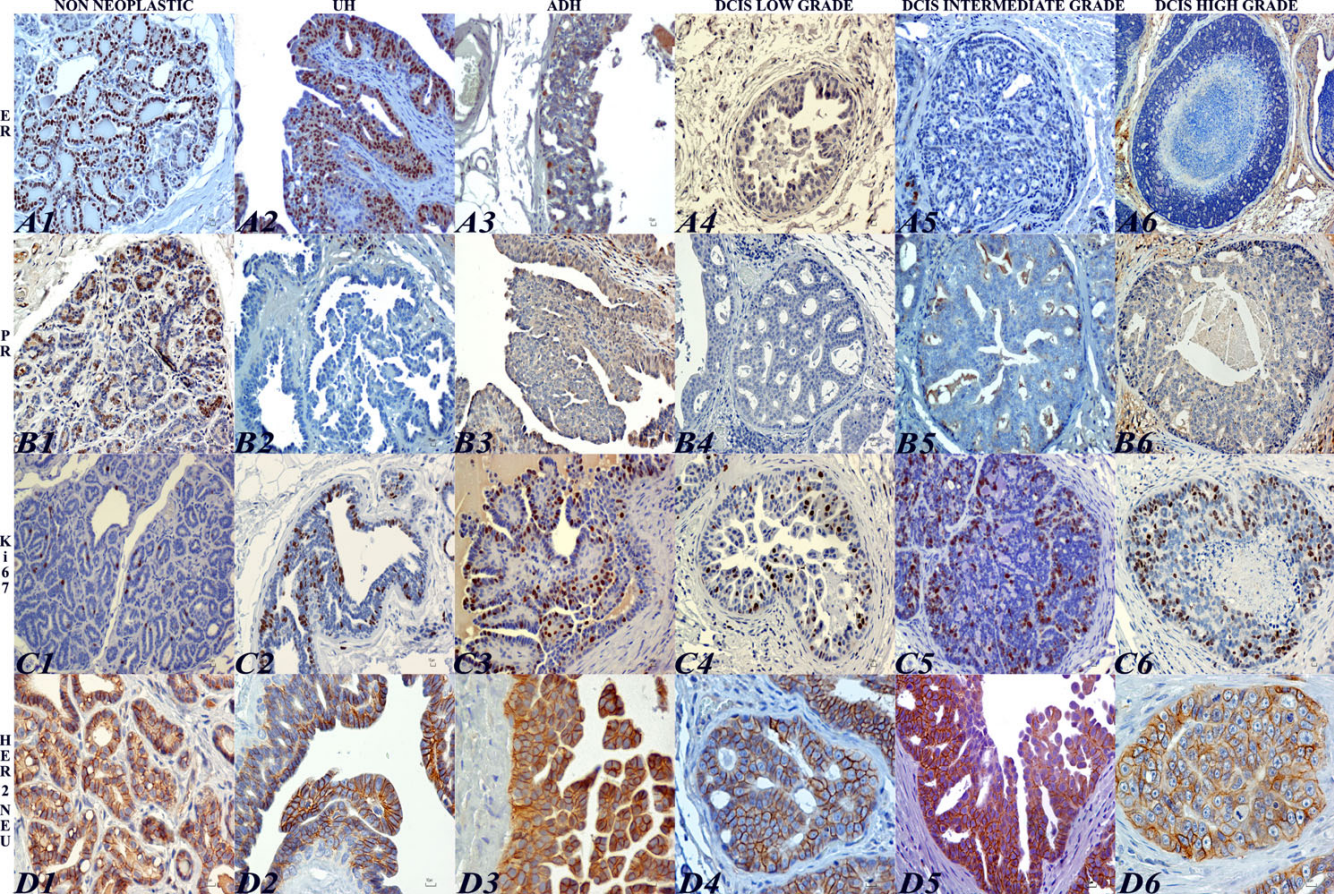
**Immunohistochemical evaluation of ER-α, PR, Ki-67, HER-2/*neu *in feline normal mammary gland, UH, ADH and DCIS**. Strong and diffuse nuclear expression of ER-α in non neoplastic feline mammary gland (A1) and in UH (A2). ADH with patchy ER-α expression in epithelial cells (A3). Low (A4) intermediate (A5) and high grade (A6) DCIS with no ER-α expression. Immunoperoxidase-DAB. Bar = 10 μm. Strong and multifocal nuclear immunoreactivity of PR in non neoplastic feline mammary gland (B1). Lack of PR immunoreactivity in UH (B2), ADH (B3), low (B4), intermediate (B5), and high grade (B6) DCIS. Immunoperoxidase-DAB. Bar = 10 μm. Few and strong Ki-67 nuclear immunoreactivity in non neoplastic feline mammary gland (C1). UH displaying strong and scattered Ki-67 immunolabeling (C2). ADH with widespread and strong Ki-67 expression (C3). DCIS low (C4), intermediate (C5), and high grade (C6) with strong and multifocal Ki-67 positive cells. Immunoperoxidase-DAB. Bar = 10 μm. Non neoplastic feline mammary gland with strong complete cell-membrane staining for HER-2/*neu*. IHC score: 3+ (D1). UH displaying moderate complete membrane HER-2/*neu *expression in few cells. IHC score: 2+ (D2). ADH showing strong complete membrane HER-2/*neu *expression. IHC score: 3+ (D3). Low-grade DCIS depicting strong complete membrane for HER-2/*neu *expression. IHC score: 3+ (D4). Intermediate-grade DCIS showing moderate complete membrane HER-2/*neu *immunoreactivity. IHC score: 2+ (D5). High-grade DCIS showing strong and complete membrane HER-2/*neu *staining. IHC score: 3+ (D6). Immunoperoxidase-DAB. Bar = 10 μm

Fifteen of 20 non-neoplastic feline mammary tissue (75%) expressed strong to moderate ER immunoreactivity in 65% of epithelial cells (mean ± SD: 65 ± 4.81; median: 64; range: 60 - 76). Immunoreactivity was also detected in 10 of 16 (62.5%) UH, in which 26% of the cells had weak to strong immunoreactivity (mean ± SD: 25.7 ± 2.1; median: 5; range: 1 - 90). Ductal hyperplasia had significantly lower ER-α expression compared to adjacent nonlesional gland (P = 0.005). Only 1 of the 15 ADH (7%) lesions had ER expression, which was of moderate intensity and detected in 5% of the cells (mean ± SD: 5 ± 1,15; median: 5; range: 3 - 5). No ER expression was detected in DCIS. Of the 44 malignant tumors examined, only 3 had weak to moderate ER expression in 8% of the neoplastic cells (mean ± SD: 8 ± 1.28; median: 8; range: 8 - 12).

#### Expression of PR in IELs (Figure 3, B1-B6)

In 2 of 20 feline mammary non-neoplastic tissue (10%), PR immunoreactivity was moderate in 17% of epithelial cells (mean ± SD: 17 ± 2.23; median: 15.5; range: 14 - 26). Weak immunoreactivity was detected in 1 of 16 UH (6%) in 5% of epithelial cells (mean ± SD: 5 ± 1.15; median: 4.5; range: 4 - 7), 1 of 15 ADH (7%) in 2% of cells (mean ± SD: 2 ± 0.67; median: 2; range: 1 - 3) and 1 of 6 DCIS low-grade (17%) in 2% of cells (mean ± SD: 2 ± 0.82; median: 2; range: 1 - 3). Expression of PR was not detected in the intermediate or high-grade DCIS lesions or in examined invasive mammary tumors.

#### Expression of Ki67 Nuclear Antigen in IELs (Figure 3, C1-C6)

Only a few normal epithelial cells had strong Ki67 expression (mean ± SD: 0.62 ± 1.06; median: 0.6; range: 0.3 - 1). Immunoreactivity for Ki67 correlated with the IEL grade, with the strongest expression in high-grade DCIS. There were significant differences among UH (mean ± SD: 2.56 ± 1.68; median: 2; range: 1 - 10), ADH (mean ± SD: 9.73 ± 2.14; median: 6; range: 1 - 25) and DCIS (mean ± SD: 9.04 ± 1.62; median: 7.5; range: 2 - 26). Specifically, Ki67 expression was significantly higher in ADH (p-value, 0.002) and DCIS (p-value, 0.002) than in UH. However, Ki67 did not differ significantly between ADH and DCIS (p-value, 0.7). Ki67 expression in IELs correlated with that in the adjacent tumors. A regression model is fitted as Ki67 in tumors (% value) = 0.038 + 0.609 * Ki67 in IEL (% value). This regression model is significant with a p-value < 0.0001. Similarly, Ki67 expression was increased in neoplasms with higher histologic grade; however, the difference was only significant between grade 3 tumors and adjacent non-lesional gland (p-value < 0.05).

#### Expression of HER-2/Neu in IELs (Figure 3, D1-D6)

The expression pattern of HER-2/*neu *antigen was unexpected. Strong and complete membrane immunoreactivity was seen in greater than 10% of the epithelial cells in non-lesional mammary gland adjacent to IELs or tumors (scored 3+). Four of 16 UH (25%) and four of 15 ADH (27%) were positive. Positive UH and ADH lesions were scored 2+, except one ADH, which was scored 3+. In DCIS, HER-2/*neu *was over-expressed in 9 of 28 cases (32%); 7 DCIS were scored 2+ and 2 DCIS were scored 3+. All remaining IELs were negative (scored 0 or 1+). Twelve of 44 neoplasms (27%) were positive; 10 tumors were scored 2+ and 2 tumors were scored 3+.

### SDS-PAGE and Western Immunoblotting

**Figure 4:**
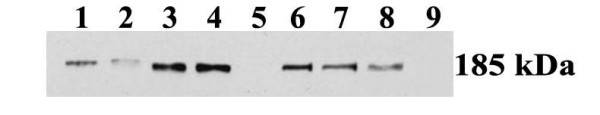
**Reactivity observed by western immunoblot with the Dako Cytomation anti-HER-2/*neu *antibody in 4 feline mammary tumors**. Lanes 1-4: Feline mammary tumors; Lanes 5-8: non-neoplastic feline mammary tissues; Lane 9: feline liver. The molecular weight of the reactive protein band is indicated on the right.

To investigate whether the IHC reactivity in non-lesional or lesional mammary tissue was due to a true expression of HER-2/*neu *or to a non-specific reactivity against other proteins, 5 normal feline mammary glands and 5 malignant feline mammary tumors were subjected to western immunoblotting. A 185-kDa band, corresponding to HER-2/*neu*, was observed in tumor samples (Figure 4, lanes 1-2-3-4). The molecular weight corresponded to that described by the manufacturer of the DAKO antibody and it was confirmed by the human protein atlas for human HER-2/*neu*. Unexpectedly, a band of corresponding molecular weight was also observed in non-lesional mammary tissue samples (Figure 4, Lanes 6-7-8). However, differences in band intensity were observed at comparable total protein loads. In particular, blotting of neoplastic tissues produced two different types of band, either of weak (Figure 4, Lanes 1-2) or strong intensity (Figure 4, Lanes 3-4). Normal mammary tissues had a clearly detectable band of intermediate intensity (Figure 4, Lanes 6-7-8). Only one sample was negative (Figure 4, Line 5); however, features of degradation in the total protein pattern might explain the negativity of that sample. Feline liver, used as a control, did not react with the same antibody (Figure 4, Lane 9).

## Discussion

Atypical lesions (ADH; DCIS) are predictors of invasive breast cancer [3,4]. However, monitoring the progression and invasion of these lesions in humans is not practical because the current standard therapy for DCIS is complete excision [35]. Thus, establishing an animal model for IELs that correlates with invasive mammary carcinoma is important to develop preventive measures and effective treatments as well as for understanding the pathogenesis of the breast cancer.

Mammary IELs have not been well characterized in genetically engineered mouse models [36], principally because they are not spontaneous, but rather induced by chemicals, radiation, or genetic modification. As in humans, and in contrast to mice and rats, spontaneous mammary tumors are quite common in cats [9,11]. Even though cats may not develop mammary neoplasia as frequently as dogs, their tumors more closely resemble those in women. For example, the benign mixed tumor that is so common in dogs almost never develops in cats or women [37].

Although feline IELs (ductal hyperplasia and carcinoma *in situ*) have been reported, these lesions were not described in detail or compared with human IELs. Consequently, we evaluated mammary IELs and expression of ER, PR, and HER-2/*neu *in feline mastectomy specimens. Ki67 proliferation index was also estimated.

IELs were observed in 28% of mastectomy specimens from female cats with clinical mammary disease; 79% were associated with malignant neoplasms. DCIS was the most common lesion, as in human mammary biopsy specimens [26]; 89% of DCIS lesions were adjacent to malignant tumors. ADH was detected less commonly than DCIS; nevertheless, 95% of these lesions were adjacent to malignant tumors. In contrast, about 50% of UH lesions were adjacent to benign tumors, duct ectasias or fibroadenomatous change, consistent with its only slightly elevated cancer risk in women [38]. In our study, the prevalence of IELs in feline mammary gland may have been underestimated because only minimal peritumoral tissue was available for histologic evaluation.

Estrogen receptor expression in benign mammary epithelium could be a risk factor for malignancy by rendering cells susceptible to the proliferative stimulus of estrogens [39]. In this study, ER was expressed in 62.5% of UH and in 7% of ADH, whereas all DCIS and 93% of tumors were negative. These data confirm that some feline mammary dysplasias and most neoplasms are estrogen receptor-negative as reported by Martin de las Mulas [20] and Millanta [40]. In cats, ER expression dramatically decreased as the IELs increased in grade; almost all neoplasms were negative for this marker. Most preinvasive lesions were ER-negative. Allred suggested that human ER-negative IELs could be involved in the development of ER-negative DCIS and its evolution into the 30% ER-negative human breast cancers [19].

PR immunoreactivity was low in non-lesional mammary gland, IELs, and tumors in contrast to the findings of Millanta and de las Mulas [40,41]. This disparity may be due to a different proportion of ovariectomized cats, different stages of the estrus cycle, administration of exogenous progestins, or different PR immunohistochemical technique. Positivity was observed in only 6% of UH, in 7% of ADH, and in 17% of low-grade DCIS. No immunoreactivity was detected in intermediate-grade or high-grade DCIS, or in any of the 44 tumors. As for ER, PR expression decreased with increasing grade of IEL.

In agreement with Millanta and Dias Pereira [42,43], the Ki67 proliferative index increased from normal mammary tissue through IELs to malignant tumors. In our study, the expression of Ki67 correlated with the grade of malignant lesions and was inversely associated with ER expression. In fact, highly proliferative lesions tended to lose ER expression. In humans, Ki67 expression increased with increasing tumor grade and correlated with decreased overall survival rates and poor response to hormonal therapy. In cats, use of Ki67 as a prognostic factor for survival with mammary carcinoma has produced conflicting results. Studies by Castagnaro *et al *revealed an association between Ki-67 index and biological behavior [44]; however, Millanta *et al *reported no significant prognostic importance in feline mammary carcinomas [42]. In a recent investigation by Dias Pereira, the Ki67 index correlated positively for different histologic lesions and tumor types with grade [43].

HER-2/*neu *IHC results were surprising and differed from those of De Maria, Ordas, and Millanta [23-25]. Those authors reported no immunoreactivity [24,25], or a faint, barely perceptible signal in part of the cell membrane [23] in normal mammary ducts and acini. A number of normal tissues, including breast, express this receptor, which probably has a role in normal cell function, regulating growth and proliferation [45]. However, we found HER-2/*neu *protein expression in normal mammary epithelium with strong, complete membrane staining (3+), contrary to what is observed in humans [46]. Immunohistochemical HER-2 protein overexpression was found in 27% of IELs and in 27% of tumors. HER-2/*neu *expression was confirmed by Western Blot, in which both normal and neoplastic tissue showed a 185 kDa band, corresponding to human HER-2/*neu*. Differences in signal intensity, however, were observed at comparable total protein loads. This result could reflect a higher expression of HER-2/*neu *in some neoplastic tissues, as in the case of samples corresponding to lanes 3-4 of Figure 4, characterized by a stronger signal compared to neoplastic samples in lanes 1-2, and to healthy tissues (lanes 5 to 8). However, the presence of HER-2/*neu *signal in adjacent histologically normal tissues, although constantly observed throughout this study, is unexpected, and needs explanation. If the DAKO antibody cross-reacts with a physiological epidermal growth factor normally expressed in the feline mammary gland, an increase in antibody specificity could overcome this issue. If the polyclonal antibody reacts with both HER-2/*neu *and another epidermal growth factor receptor (EGFR) normally expressed in ducts and acini of non neoplastic mammary feline tissue, that would explain the differences in signal intensity both within neoplastic samples, and between neoplastic and healthy tissue samples. Furthermore, the HER-2/*neu *protein could be present at higher levels in the normal feline mammary gland compared to the normal human mammary gland. Further investigations will be necessary to clarify the exact nature of this unexpected reactivity.

Similar to what was reported by Antuofermo in dogs, about half the feline IELs without atypia (UH) were associated with benign disease, whereas atypical IELs (ADH and DCIS) were generally associated with mammary cancer [47]. The histologic grades of feline mammary carcinomas in our study were similar to those reported by Castagnaro [31]. Like Seixas [34], we recognized cases of micropapillary carcinoma.

## Conclusions

In summary, mammary IELs develop spontaneously in female cats, with high prevalence, and share the full spectrum of morphologic features with human preinvasive breast lesions. The hypothetical multistep model of breast carcinogenesis proposes that invasive carcinoma arises via a series of intermediate hyperplastic lesions through various grades of atypia to *in situ *and invasive carcinoma. This implies that most ER-negative invasive breast carcinomas probably evolved from ER-negative DCIS, both of which represent about 25% of their respective categories. Similarly, most DCIS probably evolved from ADH, in which nearly all cells are highly ER-positive. However, at least a few ER-negative cells are present in all types of preinvasive lesions, including ADH, and could be progenitor cells in the development of ER-negative DCIS [19]. The loss of ER and PR expression in most feline atypical IELs and carcinomas supports the cat as a model for human ER- and PR-negative pre-invasive breast disease. New technology, such as microdissection, DNA microarray, and proteomics, will help elucidate the factors involved in the progression from IELs to invasive breast cancer.

## Competing interests

The authors declare that they have no competing interests.

## Authors' contributions

GPB: designed the study, performed the histopathological evaluation and IHC experiment and drafted the manuscript.

SIM.: coordinated, supervised and critically revised the manuscript.

MAM: performed the first histopathological evaluation and critically revised the manuscript.

VM: performed the second histopathological evaluation and critically revised the manuscript.

SP: performed the histopathological evaluation and critically revised the manuscript.

MFA: performed Western Blot experiments and critically revised the manuscript.

SU: revised the manuscript for important intellectual content.

EA: designed, coordinated, founded the study, drafted and critically revised the manuscript.

All authors read and approved the final manuscript.

## Pre-publication history

The pre-publication history for this paper can be accessed here:

http://www.biomedcentral.com/1471-2407/10/156/prepub
